# Contribution of Dynamic Rheology Coupled to FTIR and Raman Spectroscopies to the Real-Time Shaping Ability of a Hyperbranched Polycarbosilane

**DOI:** 10.3390/molecules28186476

**Published:** 2023-09-06

**Authors:** Nilesh Dhondoo, Julie Cornette, Sylvie Foucaud, Maggy Colas, Romain Lucas-Roper

**Affiliations:** IRCER, UMR 7315, Université de Limoges, F-87068 Limoges, France; nilesh.dhondoo@unilim.fr (N.D.); julie.cornette@unilim.fr (J.C.); sylvie.foucaud@unilim.fr (S.F.)

**Keywords:** polymer synthesis, rheology, vibrational spectroscopy

## Abstract

In the field of non-oxide ceramics, the polymer-derived ceramic (PDC) approach appears to be very promising, especially for obtaining easily shaped and homogeneous materials in terms of structure and composition. However, in order to reach a suitable form during the process, it is often necessary to study the rheology of preceramic polymers while they are modified during polymerisation or crosslinking reactions. Given this need in the understanding of the real-time rheology of macromolecules during their synthesis, a rheometer coupled with both an infrared spectrometer and a Raman probe is described as a powerful tool for monitoring in situ synthesised polycarbosilanes. Indeed, this original device allows one to control the viscosity of a hyberbranched polycarbosilane from defined difunctional and tetrafunctional monomers. Meanwhile, it links this evolution to structural modifications in the macromolecular structure (molar masses, dispersity and conformation), based on SEC-MALS analyses, synchronised by the monomer conversion determined by using Raman and infrared spectroscopies, a common denominator of the aforementioned instrumental platform.

## 1. Introduction

One of the goals of the field of structural material in the coming years is the development of materials operating in severe environments, such as at high temperatures (T > 1400 °C), under oxidising atmospheres. The targeted application sectors are nuclear, aeronautic propulsion or the steel industry. In these different application areas, the issues are to enhance the performance and the lightness of structural materials, reduce the fabrication cycles by decreasing the assemblage operations and improve the lifetime, performance and consumption of these systems. Non-oxide ceramics are good candidates for addressing these issues. Among them, silicon-based ceramics and composites present excellent thermomechanical properties such as high strength, hardness and low density [1]. In addition, their excellent creep, oxidation and corrosion resistance make them valuable high-temperature materials, with self-healing properties [2,3]. Since Yajima et al. reported the conversion of a polycarbosilane to silicon carbide, much attention has been focused on the structure and properties of polycarbosilanes and more generally on polymer-derived ceramics (PDCs) [4,5]. Indeed, this approach can be combined with numerous shaping processes (casting, rapid prototyping, aerosol spraying, etc.). Moreover, the structures of these polymers influenced the chemical composition, ceramic yield, oxidation resistance and mechanical properties of the resulting ceramics [6]. Comprehensive knowledge of the synthesised polymers is the best way to understand and predict its shaping and thermal behaviour. This involves the use of controlled polymerisation reactions, in order to obtain well-defined microstructures of the materials during the ceramisation of the precursor.

In order to establish a link between polymer architectures and the effect on future ceramics, a crucial point is to perform the most complete monitoring of the polymerisation reaction. This requires real-time control of the construction of macromolecular structures under defined atmosphere and temperature. In addition, the rheological data of the polymer are of great importance because they will condition their future shaping. To answer this need to retrieve multiple parameters at the same time, which will improve the quality of production of polycarbosilanes, adapted instrumentation is necessary. Since one of the challenges of the preceramic precursor strategy is controlling physical and chemical transformations that occur during polymerisation, a device that dynamically combines a rheometer and an infrared spectrometer could be useful for monitoring the structure of the growing macromolecules and their rheological behaviour at the same time. Several studies have used a rheometer coupled with a Fourier-transform infrared (FTIR) spectrometer, dynamically combining a rheometer with an infrared spectrometer. Maia et al. were the first to report this dynamic coupling, by studying the crosslinking of a vinylester resin and proving the interest in the in situ FTIR measurements, in comparison with the ex situ method, for such systems [7]. Since then, two other studies have been published, first, on a reversible Diels–Alder crosslinking reaction in a polymer network, and secondly, on the improvement of the thermal and rheological behaviours of poly(3-hydroxybutyrate) by adding tannic acid [8,9]. More recently, we reported the use of a rheometer coupled to an IR spectrometer, to monitor the polymerisation of hyperbranched polycarbosilanes [10,11]. In addition to the interesting results obtained by this coupling device in terms of polymerisation kinetics and rheological behaviour, a difference in the polymer growth was assumed between the wall (where the diamond window of the ATR detector is positioned) and inside the sample.

To understand these differences, the rheometer could also be equipped with a fibered Raman spectrometer, known as a powerful tool for characterising the local structure of materials and providing a greater depth of penetration compared to ATR in analysed materials [12,13,14] when used with lens instead of objective. Indeed, even if they are vibrational spectroscopies, they provide complementary structural information. Several studies have been published on the coupling of a rheometer with a Raman spectrometer. Among them, Chevrel et al. used a laboratory-made rheo–Raman device [15], in order to monitor free-radical polymerisation of acrylic acid in an aqueous medium. This was carried out by following either the band at 1635 cm^−1^, corresponding to a stretching vibration mode of the double bond C=C of the monomer, or the asymmetric stretching vibration mode of the C–H bond at 2935 cm^−1^ characteristic of the formation of poly(acrylic acid). The mixer-type rheometer was equipped with a quartz outer cylinder, and it was combined with a Raman spectrometer. Using a Couette analogy, different parameters were accessible at the same time, such as the monomer conversion, polymer formation and viscosity of the medium. This coupling also allows the authors to monitor styrene polymerisation and develop a high-impact polystyrene process [16,17]. This time, the authors decided to follow the intensity of the band at 1630 cm^−1^ attributed to the vibrational mode of the C=C bond of the vinyl group. Another way to measure a Raman signature is to employ a rotational rheometer coupled to a Raman microscope through an optically transparent modified base [18]. This arrangement was used to study the melting transition of cosmetic emulsion, or the crystallisation of a high-density polyethylene.

**Figure 1:**
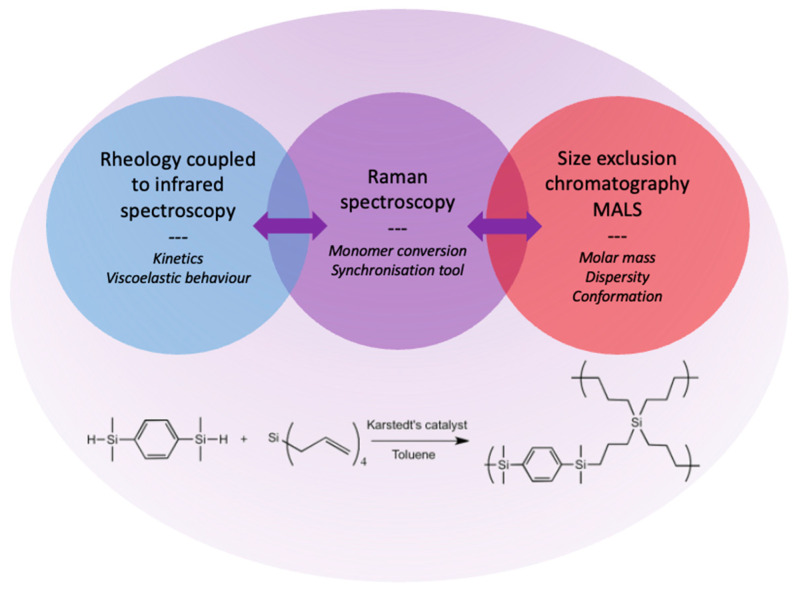
Schematic representation of the instrumentation and of the polymerisation reaction leading to the hyperbranched polycarbosilane (*hb*-PCS).

Based on these promising results from the literature, and in order to develop an original coupling device, a fibered Raman spectrometer is combined with the rheo–FTIR instrumentation in this article (Figure 1). As it is not possible to collect the polymerisation medium using the rheometer, the fibered Raman spectrometer also serves as a synchronisation tool in another part of the setup, by probing the same polymerisation reaction. Indeed, another key characterisation concerns the molar masses of the new generated macromolecules, to understand their growth and create models coupling rheology and macromolecular structures in bulk syntheses. To achieve this and to create a unique, coherent and polyvalent platform of analysis, the use of size exclusion chromatography (SEC) with a multiangle light scattering detector (MALS) is selected. Indeed, this instrumentation allows one to access vital information to monitor the polymerisation of carbosilanes towards a hyperbranched polycarbosilane (*hb*-PCS) (Figure 1). In this paper, this unique multiparameter platform is described as a powerful tool for carrying out experiments on polymerisation with both a structural and shaping perspective of the growing preceramic materials, from functionalised carbosilanes.

## 2. Results and Discussion

### 2.1. Rheology Coupled to Raman and Infrared Spectroscopies

#### 2.1.1. From Spectroscopic Signatures

**Figure 2:**
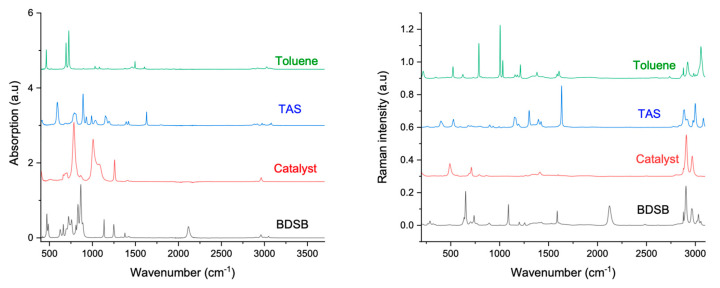
IR and Raman spectra for reagents.

Before considering monitoring the polymerisation reaction, the spectroscopic signatures of the reagents must be considered (Figure 2). In this way, the wavenumber range from 1500 to 2300 cm^−1^ is of particular interest as characteristic bands of the monomers are found in this region and they did not overlap each other. It was noticed that the stretching vibration of C=C from TAS is present in both IR (1630 cm^−1^) and Raman spectroscopy (1632 cm^−1^). Furthermore, it was observed that the stretching vibration of Si-H is present in both IR (2117 cm^−1^) and Raman spectroscopy (2122 cm^−1^). A deeper vibrational analysis (FTIR and Raman) of the BDSB molecule was performed because of a difference between both Raman and IR spectra; in fact, a stretching vibration of C=C_ar_ seemed to be absent from the FTIR spectrum (Figure 2). As the symmetry of the molecule is probably the origin of this difference, an ab initio calculation was undertaken to highlight this point, which to our knowledge has never been highlighted in the literature.

Because of the geometry of the molecule BDSB, it can be described as a C2h symmetry. Based on the C2h character table, active modes in Raman are inactive in FTIR, hence verifying the principle of mutual exclusion for all molecules which have an inversion centre.

**Figure 3:**
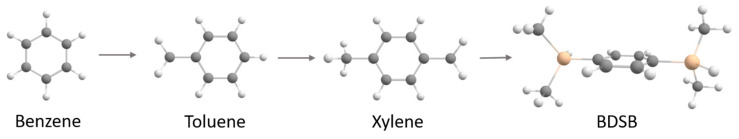
From benzene to BDSB: a question of symmetry.

Then DFT calculations were carried out to demonstrate the effect of a centre of inversion on the vibration modes of molecules. They were performed on optimised geometries to determine the vibrational frequencies and IR and Raman intensities. To follow the evolution of the vibration of C=C_ar_ function, calculations started from the basic molecule of benzene, which is the skeleton of the BDSB molecule. Then, the molecule was turned into an asymmetric molecule by adding a CH_3_ function. By connecting a second CH_3_ function, the symmetry of the molecule was restored. Finally the CH_3_ functions were replaced by [SiH(CH_3_)_2_]_2_ functional groups to construct the BDSB molecule. In this way, the evolution of the calculated C=C_ar_ frequencies was followed in both Raman and IR spectra. Figure 3 shows the construction from the benzene molecule up to the BDSB molecule. The molecule of xylene is close to the BDSB molecule in terms of symmetry; however, due to the difference in weight (cf. [SiH(CH_3_)_2_]_2_), their frequencies will be different.

**Figure 4:**
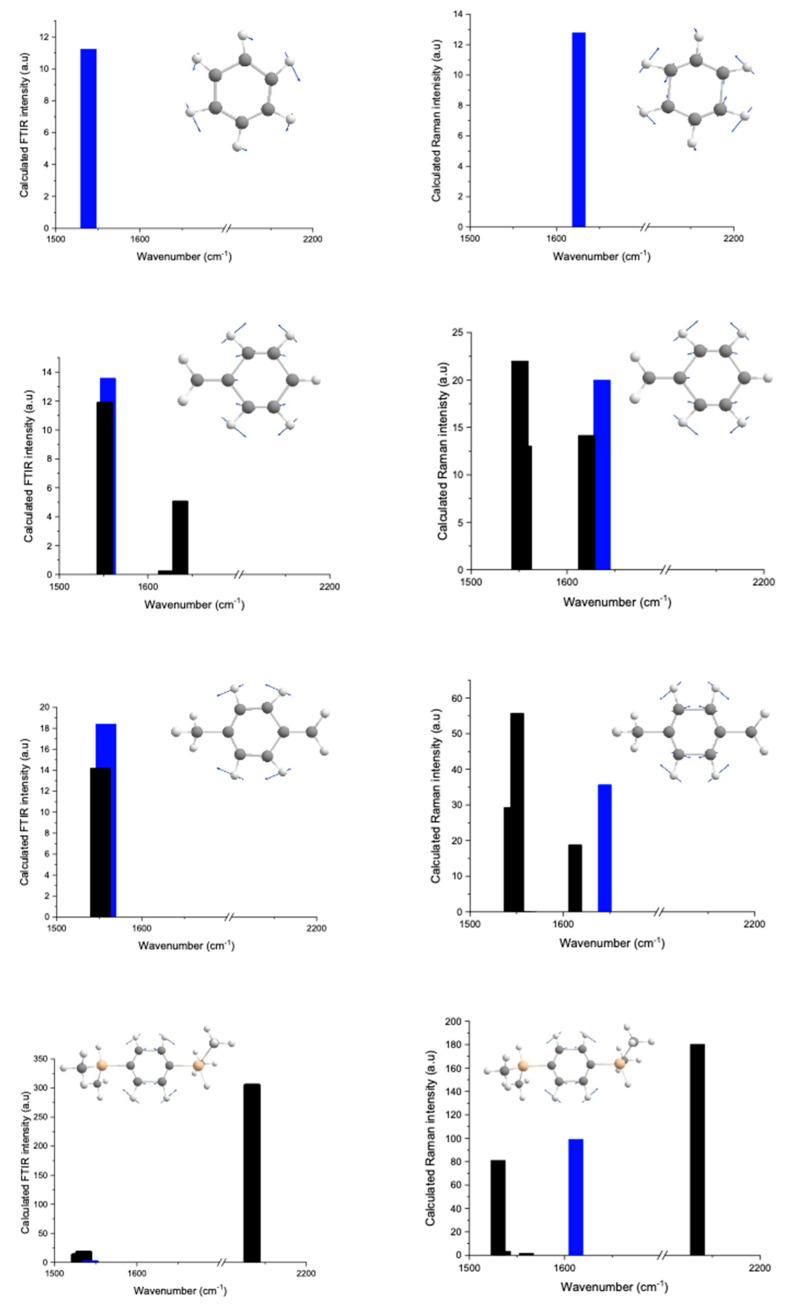
Calculated FTIR and Raman intensities obtained by DFT for four molecules: benzene, toluene, xylene and BDSB. The stretching vibration of C=C_ar_ was marked in blue colour.

Figure 4 reports the different frequencies of the vibrational modes obtained for Raman and FTIR by DFT calculation of benzene, toluene, xylene and BDSB in the region from 1500 cm^−1^ to 2300 cm^−1^. In FTIR DFT, the stretching vibration of C=C_ar_ in benzene was identified at around 1539 cm^−1^, while in Raman DFT, it was reported at 1626 cm^−1^. By knowing the initial frequency of C=C_ar_, its evolution can be followed in the other molecules. Figure 4 particularly highlights the frequency evolution of C=C_ar_ in each molecule and the stretching vibration of C=C_ar_ that could be distinguished from the rest as it was marked in blue colour. Thus, for both Raman and FTIR, the initial frequency value of C=C_ar_ reported in benzene was different in toluene, xylene and BDSB molecules. Therefore, to successfully follow the evolution of C=C_ar_, the vector displacements of C=C_ar_ have to be considered to identify this frequency in each molecule. In FTIR DFT, the calculation of the BDSB molecule showed a very weak intensity of C=C_ar_ frequency at 1564 cm^−1^, while in Raman DFT, a strong intensity was found for C=C_ar_ at a frequency of 1626 cm^−1^. The intensity of the frequency of C=C_ar_ in FTIR DFT was four orders of magnitude lower in comparison to the Raman DFT; hence, it appeared negligible. The strong intensity of the Si-H band was reported in both FTIR and Raman DFT at around 2134 cm^−1^. Consequently, the DFT results of the BDSB molecule was in accordance with the Raman and FTIR experimental results, i.e., due to the symmetry and the principle of mutual exclusion, there was an absence of a C=C_ar_ vibration. Thus, for the rest of the experiment and data treatment, the band of C=C from the alkene in TAS is discussed.

#### 2.1.2. To the Optimisation of a New Coupling Instrumentation

**Figure 5:**
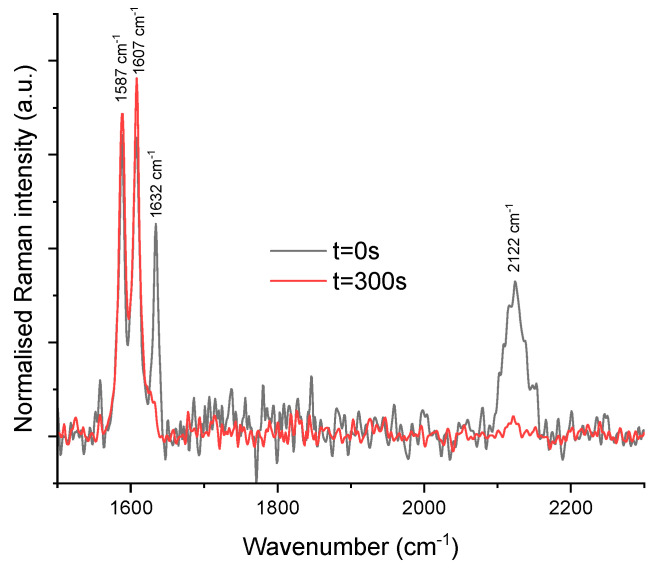
Raman spectra of the starting mixture (black line) and of the resulting polymer solution (red line) obtained from the rheo–FTIR–Raman setup.

The challenge of using the Raman spectrometer with a lens of focal distance of 100 mm lay in successfully probing the sample completely with the selected 600 μm gap distance. Indeed, it was a strenuous task to probe the sample given the restriction imposed in terms of gap. After several attempts, and by modifying the position of the lab-made Raman support, it appeared that the signal-to-noise ratio was minimised when the 100 mm focal distance lens was well positioned with respect to the plate–plate geometry (Figure S1). From the region of interest (from 1500 to 2300 cm^−1^), the triplet bands representing the stretching vibration of C=C of BDSB at 1587 cm^−1^, C=C of toluene at 1607 cm^−1^ and C=C of TAS at 1632 cm^−1^ together with the stretching vibration of Si-H at a higher wavenumber (2122 cm^−1^) are distinctly illustrated in Figure 5. Consequently, the Raman data obtained from the coupling were exploitable and could be used to calculate the monomer conversion. Moreover, as expected, the stretching vibration of the C=C in TAS as well as the stretching vibration of Si-H were consumed during the polymerisation reaction.

To check the homogeneity of monomer conversion across different positions of the geometry [10], an investigation was performed by varying distances across the radius of the plates, namely, **P0**, **P1** and **P2**, at 0, 5 and 10 mm, respectively (Figure S2). A time-dependent oscillatory test was carried out with a frequency set at 35 Hz and a strain at 0.5%. The different alkene conversion at positions **P0**, **P1** and **P2** calculated from the FTIR data showed that the same trend was obtained and a good superposition was noticed until a monomer conversion of 0.58, characteristic of the gel point of the system [11]. Henceforth, the monitoring of the hydrosilylation reaction carried out at different positions indicated that the same kinetics occurred across the radius of the geometry. This finding is in accordance with a study carried out by Plog et al., who reported insignificant differences in crystallinity of polyethylene at different points from the rim of the plate to the centre determined by the rheo–Raman microscope setup [18].

**Figure 6:**
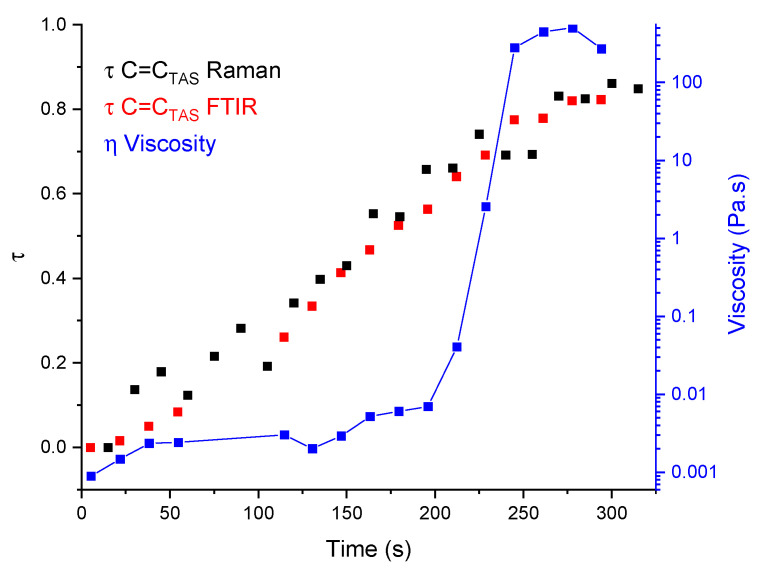
Coupling results of the rheo–Raman–FTIR in a PP configuration.

In order to match the mixing of the reaction medium by the magnetic stirrer in RBF, the rheological tests were carried out in a steady-state experiment at 4.5 s^−1^ to be compared to the frequency of rotation of the magnetic stirrer. After optimising the rheo–Raman–FTIR setup in terms of gap, intensity of the Raman signal, impact of catalyst concentration (Figure S3) and homogeneity of the reaction across the geometry, an experiment was carried out to monitor in real time the hydrosilylation reaction, by acquiring both infrared and Raman signatures. The coupling results of the polymerisation reaction are shown in Figure 6. The alkene conversion calculated from Raman and FTIR data showed an identical trend confirming that the same reaction kinetics occurred at the lower surface of the ATR and at the centre/rim of the sample, validating the fact that the reaction occurring across the plate–plate configuration was homogeneous. Furthermore, as expected, the viscosity remained constant during the initial moments, before increasing rapidly to reach a maximum viscosity of roughly 120 Pa s, this rapid change of viscosity was attributed to a state change from liquid to a solid gel. It was observed that the macromolecule network had an influence on the rheology of the polymer only after the calculated critical conversion of 0.58. To understand the impact of the polymer structure on its rheology, monitoring macromolecular structures (average molar mass, conformation, etc.) as a function of time would provide important additional information, and would help to reinforce the link between the shaping ability of preceramic precursors and the monitoring/control of the polymerisation.

### 2.2. Kinetics of the Hyperbranched Polymerisation through SEC-MALS Analyses

**Figure 7:**
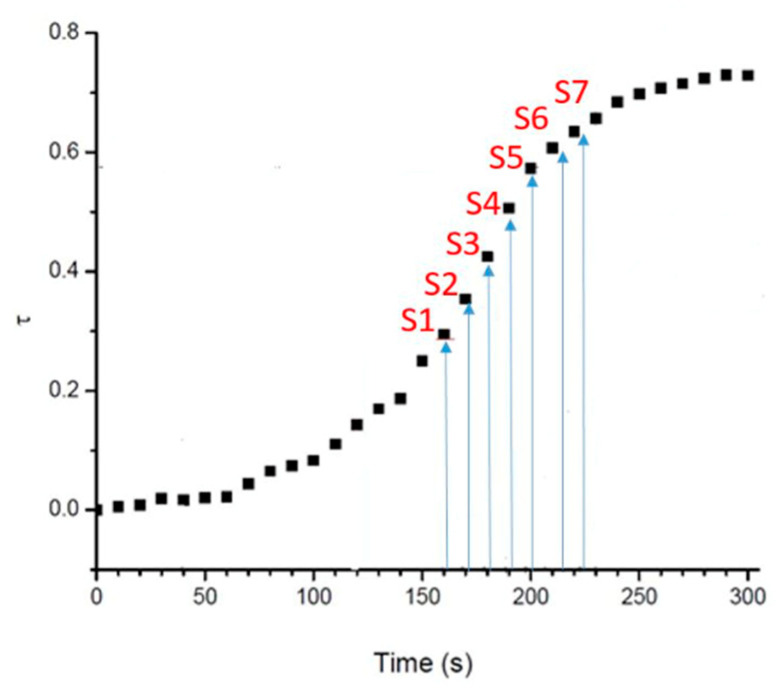
Sampling window from graph plotting the alkene conversion versus time.

The first part of this study concerns the optimisation of the sampling window, which required a sufficiently concentrated solution of monomer to produce a sufficient signal in MALS analysis but not too high, in order to avoid a too-rapid reaction. As a result, a total number of seven points was collected, named **Si** (**i** from 1 to 7, Figure 7). By the time the eighth sampling was performed, the gel was already in its solid form.

**Figure 8:**
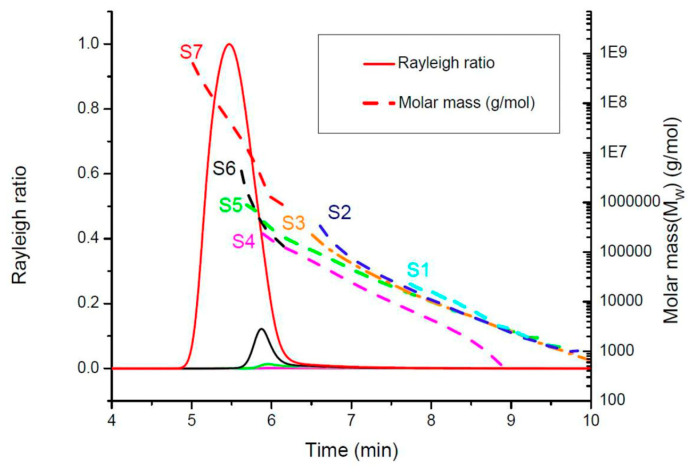
SEC elution pattern plots of the samples (**S1**–**S7**) versus time.

Figure 8 displays the elution time and the molar mass of the seven sampling solutions. The separation of the molecules was carried out by an SEC based on size (hydrodynamic radius). It could be observed that **S1** had the lowest molar mass ranging from 10^2^ to 10^4^ g∙mol^−1^ with a retention time of around 8 min, while the highest molar mass was noticed in **S7** ranging from 10^5^ to 10^8^ g∙mol^−1^ with a retention time of 5.5 min. Moreover, **S1** possessed mostly oligomers as **S1** was sampled during a stage of the step-growth polymerisation reaction where the structure of the macromolecules was not developed enough. As the polymerisation reaction progressed, the molar mass registered for **S2**–**S4** did not differ too much from each other as they were all in the range of 10^2^ to 10^5^ g·mol^−1^. The sampling solutions **S2**–**S4** were mostly macromolecules that were still under construction. Furthermore, **S5** possessed molar mass ranging from 10^3^ to 10^6^ g·mol^−1^ with the first visible peak on the chromatogram having a retention time of 5.9 min. In addition, **S5** revealed the intensification of the crosslinking of the macromolecules, thus representing the progression of the polymerisation reaction towards a fully formed hyperbranched polymer. **S6** had mostly large macromolecules with molar mass varying from 10^4^ to 10^7^ g·mol^−1^ with a retention time of 5.8 min. Finally, **S7** registered the highest molar mass with a sharp and intense chromatogram signal and a retention time of around 5.5 min. In addition, it is worth mentioning that the quenching of the polymer reaction was efficient as the same dispersion was observed one hour after the first analysis of the different samples.

**Figure 9:**
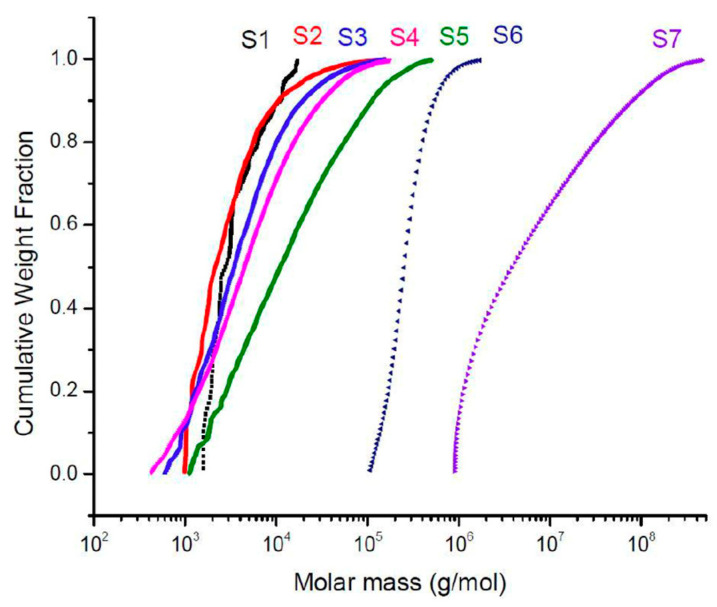
Cumulative molar mass distribution curve.

By focusing on the cumulative molar mass distribution of the collected samples (Figure 9), **S1** displayed most of its weight fraction polymer within the domain of 10^3^ to 10^4^ g·mol^−1^ with a maximum of roughly 17,500 g∙mol^−1^ and a minimum of around 1600 g·mol^−1^. The cumulative molar mass distribution of **S1** indicated a moderate molar-mass dispersity (*Ɖ_M__1_* = 1.56, Table 1), thus confirming that at around 163 s, oligomers were being formed. **S2**–**S4** had most of their cumulative molar mass distribution ranging from 10^3^ to 10^5^ g∙mol^−1^, with molar-mass dispersity increasing (*Ɖ_M_*_2_ = 2.87, *Ɖ_M_*_3_ = 3.86 and *Ɖ_M_*_4_ = 5.37). However, even if the macromolecules were in continuous development, they were moderately interconnecting with each other. Meanwhile, **S5** represented a cumulative molar mass that started above 10^3^ g∙mol^−1^ and stretched until 10^5^ g∙mol^−1^. **S5** was sampled at 206 s, and according to its molar-mass dispersity (*Ɖ_M_*_5_ = 7.81), macromolecules present at that moment possessed a wide range of molar mass. Moreover, **S6** surprisingly recorded the smallest molar-mass dispersity (*Ɖ_M_*_6_ = 1.35) with a domain of molar mass from 10^5^ to 10^6^ g∙mol^−1^. This sudden decrease in molar-mass dispersity could be explained by the fact that all macromolecules at this moment had approximately the same range of molar mass before starting to interlink with each other. Finally, the last sample point **S7** had the broadest domain of cumulative molar-mass distribution varying from 10^6^ to 10^8^ g∙mol^−1^. In addition, it also had the largest molar-mass dispersity (*Ɖ_M_*_7_ = 11.48), implying that the macromolecules were being connected to their neighbours, forming newer larger macromolecules with significantly different sizes.

**Figure 10:**
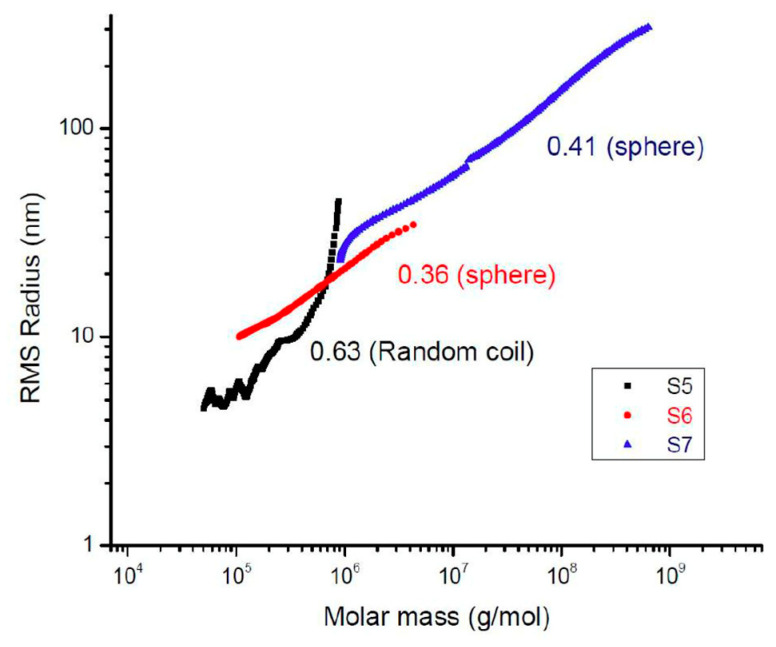
Conformation plots of samples **S5**–**S7**. The slope values are indicated for each regression.

During the SEC-MALS analysis, simultaneous measurements of RMS (Root Mean Square) radius and molar mass gave extra information about the polymer chain conformation by plotting the log of RMS radius versus log of molar mass as shown in Figure 10. **S1**–**S4** were not represented since their RMS radii were less than 10 nm. The slope of the conformation plot of the molar-mass dispersed samples could be classified into three categories: (i) rod with a slope = 1, (ii) random coil with slope between 0.5 and 0.6, (iii) sphere (characteristics of hyperbranched polymer) with slope between 0.3 and 0.4 [19,20]. Random coil structures are typically an indication of linear polymers, whereas sphere-like molecular structures are evidence of hyperbranched molecules. According to Figure 10, **S5** was classified as a random coil conformation with a plot of 0.63, while **S6** and **S7** were considered as spheres (hyperbranched structures) since their conformation plot slopes were 0.36 and 0.41, respectively.

### 2.3. Linking Polymer Structure to Rheology, towards a Shaping Ability

**Figure 11:**
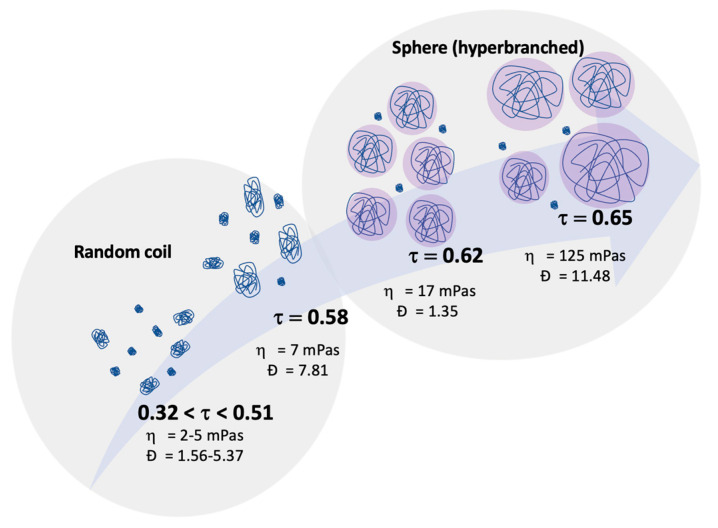
Structure/conformation of the samples **S1**–**S7** along with their average molar mass, molar-mass dispersity, alkene conversion and viscosity.

Achieving the synchronisation of the reaction kinetics through the monomer conversion, by using a fibered Raman spectrometer, it was possible to link the viscosity of the reaction medium with the average molar mass of the collected samples. It was noticed that the average molar mass and the viscosity increased gradually from an alkene conversion in the range of 0.32 to 0.65 (Figure 11). The transition between random coil structures to hyperbranched structures occurred at a critical alkene conversion of 0.58 where the macromolecular networks started to strongly influence the viscosity of the reaction medium. At an alkene conversion of 0.62, most of the active sites during the polymerisation reaction were consumed. In addition, the molar-mass dispersity recorded a sudden decrease since the macromolecules present at this moment of the polymerisation reaction were approximately in the same molar mass range.

Finally, at an alkene conversion of 0.65, the molar mass, molar-mass dispersity, and viscosity had a significant increase seconds before the gel turned into a solid form. Moreover, at this alkene conversion, the macromolecules with varying molar mass 10^6^–10^8^ g·mol^−1^ made up the hyperbranched structures of polycarbosilanes.

**Figure 12:**
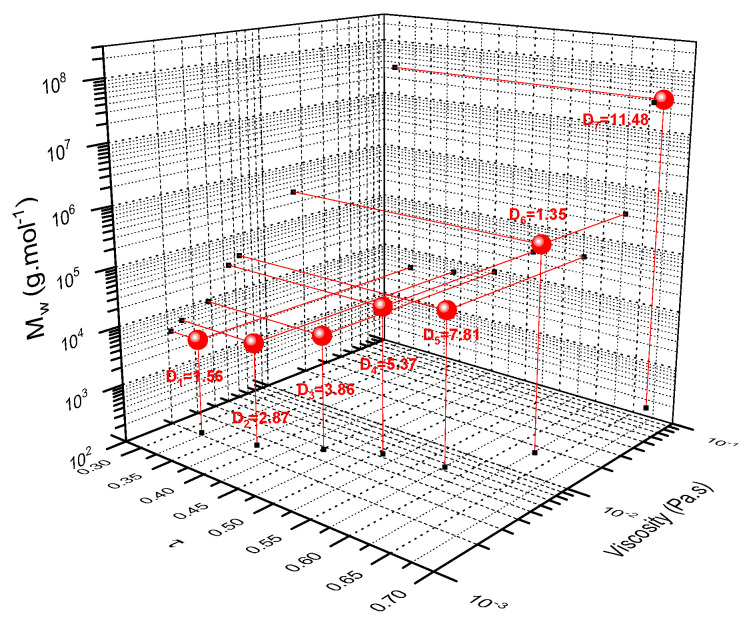
3D representation of alkene conversion plotted against average molar mass and viscosity of the medium.

Figure 12 presents a 3D graph of alkene conversion against average molar mass and the viscosity during the polymerisation reaction. As expected, the molar mass and the viscosity of the sample increased drastically only after the critical alkene conversion (0.58). Based on these combined results, a viscosity window ranging from 2 to 125 mPa∙s was established. Moreover, within this period of time, the polymerisation process can be stopped at any given moment by using a suitable viscosity that corresponds to a particular shaping process.

## 3. Materials and Methods

### 3.1. Materials

The hb-PCS was prepared using 1,4-bis (dimethylsilyl)benzene (BDSB, C_6_H_4_[SiH(CH_3_)_2_]_2_; Merck, 97%), tetraallylsilane (TAS, C_12_H_20_Si; Merck, 97%), Platinum(0)-1,3-divinyl-1,1,3,3-tetramethydisiloxane complex (C_8_H_18_OPtSi_2_, Merck, 98%) and toluene (Merck, 99.8%) as starting materials. All these compounds were commercially available and used as received.

### 3.2. Characterisations

#### 3.2.1. Rheometer Coupled to FTIR Spectroscopy

The rheological behaviour of monomer blends was studied on a rotational rheometer (Mars III, Thermo Scientific, Karlsruhe, Germany, Rheowin 4.91.0021 software), using a 35 mm plate–plate (PP) geometry. This apparatus was coupled with an infrared spectrometer (Nicolet IS10, Thermo Scientific, Omnic Series software, https://www.thermofisher.com/order/catalog/product/INQSOF018 (accessed on 29 August 2023)). A diamond window is present at the lower part of the plate–plate geometry for the laser beam to pass through it. The steady-state dynamic viscosity was determined using a shear rate of 4.5 s^−1^ at 25 °C. In addition, the rheological properties were measured in strain-controlled oscillatory tests: the frequency was set at 1 Hz or 35 Hz, and a strain amplitude of 0.5% was selected, to ensure a linear regime of oscillatory deformation.

#### 3.2.2. Raman Spectroscopy

The Raman measurements were carried out by using a RXN1 spectrometer (Kaiser Optical System) with a laser excitation wavelength at 785 nm. On the one hand, the rheo–Raman–FTIR setup uses and space constraints forced us to use a lens with a focal distance of 100 mm to perform measurements. On the other hand, the measurements were realised with a Raman immersion probe immersed in the reaction of synthesis for the MALS setup.

#### 3.2.3. Size-Exclusion Chromatography/Multiangle Light Scattering and Sampling

The molar mass/conformation analysis of the polymer solutions were carried out using a size-exclusion chromatography system from Shimadzu (LC20AD pump, CTO-20AC oven and SPD-20A detector), coupled to a DAWN HELEOS II detector (MALS) with an Optilab T-rEX refractometer, both from Wyatt. The solvent used was toluene, and the dn/dc of 0.0592 mL g^−^^1^ was determined beforehand. The sampling process was optimised during the in situ polymerisation reaction. The sampled polymer solution was immediately quenched by inserting 300 μL polymer solution sampled in a 10 mL graduated flask containing about 9 mL of toluene, until a total volume of 10 mL was reached. Sampling could be performed solely for 230 s, as beyond that point, a solid gel was formed, in sampling every 10 s, occurring in a window time of 160–230 s.

### 3.3. Synthesis

#### 3.3.1. Classic Synthesis

The polymerisation procedure in a round-bottom flask (RBF) consisted of mixing 369 μL of TAS (319 mmol L^−1^), 739 μL of BDSB (664 mmol·L^−1^) and 3.5 mL of toluene in the RBF. The addition of 385 μL catalyst solution with an optimised concentration of 0.415 mmol L^−1^) started the polymerisation reaction. In the meantime, the Raman acquisition was started when the catalyst was added to the reaction mixture.

#### 3.3.2. In Situ Monitored Synthesis

The polymerisation procedure on the rotational rheometer (Mars III, Thermo Scientific) consisted of mixing 73.8 μL of TAS (319 mmol L^−1^) and 148 μL of BDSB (664 mmol L^−1^) in a test tube, with 702 μL of toluene. At the same time, the rheometer was set in terms of inertia and gap. The addition of 77.1 μL catalyst solution with an optimised concentration of 0.415 mmol L^−1^ in a test tube started the reaction. Within seconds, the mixture was loaded in between the plates of the rheometer. The rheological, Raman and FTIR data acquisitions were initiated once the mixture was placed in between the plates of the rheometer.

#### 3.3.3. Monomer Conversion Calculation

For both Raman and FTIR, the stretching vibration band of alkene C=C in TAS was normalised by using the stretching vibration of the C=C_ar_ band of toluene since it remained unchanged during the reaction. Only the alkene conversion was calculated since, in FTIR, the amplitude of the Si-H band was influenced by the diamond crystal. Therefore, the alkene conversion versus time was calculated based on the fitted normalised peak intensity function of *t* = 0 and a time *t*, denoted *N*_0_ and *N*_*t*_, respectively. In this way, the conversion can be expressed as

$$\tau \left(t\right)=\frac{N_{0}-N_{t}}{N_{0}}\;$$

### 3.4. Computational Details

The calculations were performed within hybrid functional approximation to density functional theory (DFT) as implemented in Gaussian03 software packages. The ab initio calculations were realised in Beck’s three-parameter hybrid method using the Lee–Yang–Parr correlation functional B3LYP. This technique, being run within the 6–21 G basis set by the GAUSSIAN program, has made it possible to obtain optimised geometries and IR and Raman vibrational spectra.

## 4. Conclusions

**Figure 13:**
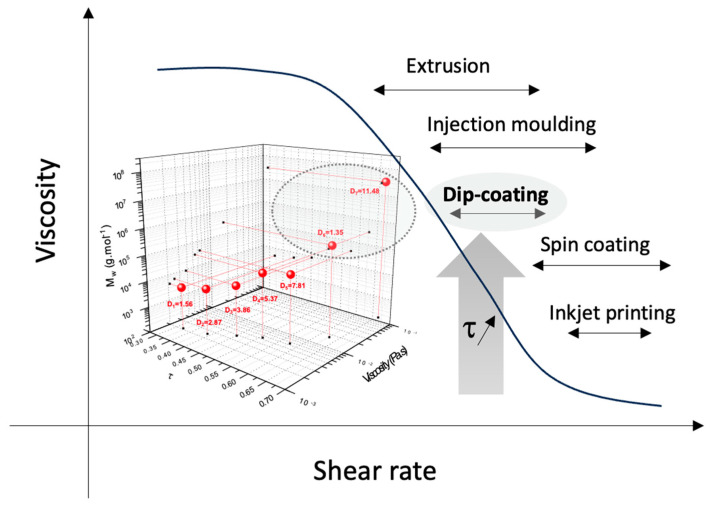
Shaping applications of the hyperbranched polymer on a graph plotting the viscosity as a function of the shear rate with [21].

The real-time monitoring of a hyperbranched polycarbosilane was successfully reported by combining infrared and Raman spectroscopies, and by linking the rheological data to the structural and conformational aspects of the in-process polymer. According to the literature, the viscosity domain obtained could correspond to a shaping process such as dip coating (Figure 13) [21]. Thus, it was possible to attribute the viscosity of the preceramic precursors to a shaping ability, which is a crucial step in the ceramic processes. This original platform would definitely be a powerful tool for adapting/controlling the viscosity of polymer solutions to a corresponding shear rate required to carry out the shaping process at optimum conditions. Extra macromolecular systems such as sol–gel structures, doped or not, are currently being investigated, with potential fallouts in the various fields implying shaping of polymers.

## Figures and Tables

**Table 1:** Average molar mass and molar-mass dispersity of the different polymer solution samples.

Sample	$\bar{Mw}$/g·mol^−1^	*Ɖ* *_M_* */-*
**S1** ^1^	4386	1.56
**S2** ^1^	5885	2.87
**S3** ^1^	8984	3.86
**S4** ^1^	31,080	5.37
**S5** ^1^	40,230	7.81
**S6** ^1^	320,200	1.35
**S7** ^2^	28,200,000	11.48

^1^ Zimm fit model; ^2^ Berry fit model.

## Supplementary Materials

The following supporting information can be downloaded at: https://www.mdpi.com/article/10.3390/molecules28186476/s1, Figure S1: (a) Photo of the rheo–FTIR–Raman in a plate–plate configuration; (b) Laser beam probing the sample through the 0.6 mm gap; Figure S2: (a) Representation of the different studied positions P0, P1, P2 and P3; (b) Monomer conversion obtained for P0, P1 and P2 from FTIR data (results obtained from P3 were disregarded due to the position of the diamond window, which was at the limit of the geometry of the plate–plate configuration); Figure S3: Effect of the concentration of catalyst on the polymerisation reaction. Successive delays of 514 s, 629 s and 603 s were reported between each catalyst concentration variation determined for a reached viscosity of roughly 10 Pa.s.
