# Supplementary material for: Contribution of Dynamic Rheology Coupled to FTIR and Raman Spectroscopies to the Real-Time Shaping Ability of a Hyperbranched Polycarbosilane
Source: Molecules. 2023 Sep 6;28(18):6476. doi: 10.3390/molecules28186476 (PMC10538020; doi:10.3390/molecules28186476)
Supplement: Supplementary file 1 [file molecules-28-06476-s001.zip › molecules-2568175-supplementary.pdf]

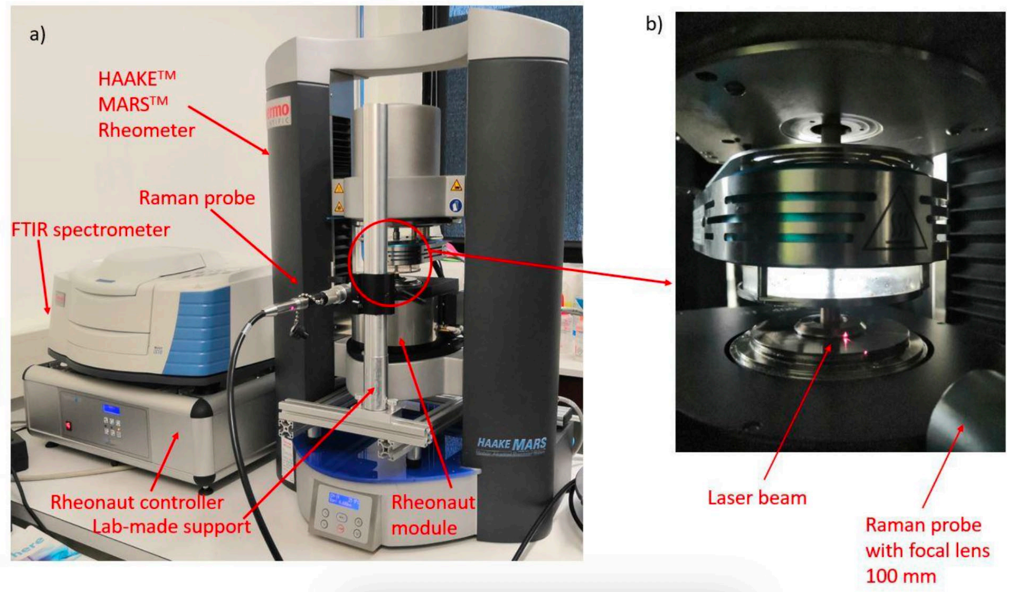

Figure S1. (a) Photo of the rheo-FTIR-Raman in a plate-plate configuration; (b) Laser beam probing the sample through the 0.6 mm gap.

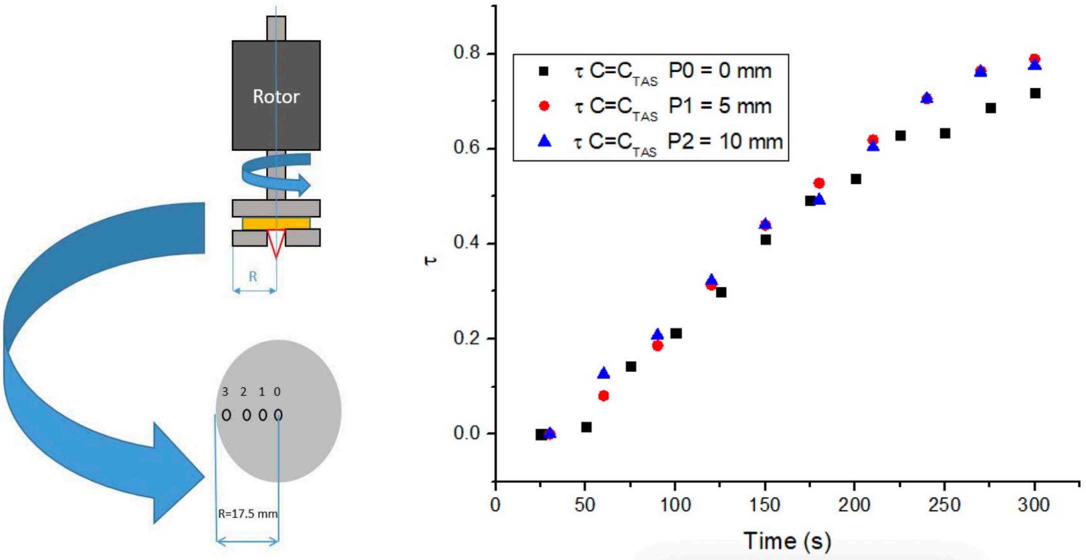

Figure S2. (a) Representation of the different studied positions P0, P1, P2 and P3; (b) Monomer conversion obtained for P0, P1, and P2 from FTIR data (results obtained from P3 were disregarded due to the position of the diamond window which was at the limit of the geometry of the plate-plate configuration).

During the initial coupling studies carried out, it was observed that the gel turned into a solid state within 230 s for a catalyst concentration of 0.415 mmol·L^{-1}. This was further demonstrated by the sudden rise of the viscosity indicating a state change occurred during the polymerisation reaction. By considering that the same outcomes would occur in RBF, it implied that sampling process could take place until around 230 s (the moment when the gel turned into a solid state). With the goal of increasing the reaction time of the polymerisation reaction as well as decreasing the effect of the sudden rise of viscosity, an investigation on the impact of different catalyst concentrations (**C1** = 0.415 mmol·L^{-1}, **C2** = 0.021 mmol·L^{-1}, **C3** = 0.017 mmol·L^{-1}, and **C4** = 0.014 mmol·L^{-1}) on the polymerisation reaction was carried out in a steady state test with a shear rate set at 35 s^{-1} (Figure S3). For each catalyst concentration, the derivative of the viscosity as a function of time was calculated to find out the maximum viscosity reached. For **C1**, **C2**, **C3** and **C4**, a maximum viscosity of 5.54 Pa·s, 12.47 Pa·s, 9.88 Pa·s and 8.31 Pa·s was

reported after a time of 300 s, 814 s, 1443 s and 2046 s respectively. Thus, it was noted that a decrease in the catalyst concentration resulted in an increase of the reaction time of the polymerisation while the maximum viscosity reached was within the same range each time for **C1**-**C4**. Therefore, although the decrease in catalyst concentration did increase the reaction time of the polymerisation, the liquid-solid gel transition time was the same in all cases - independent of the catalyst concentration. Consequently, lowering the catalyst concentration would not result in increasing the sampling process window in RBF since the catalyst concentration did not influence the sudden increase of viscosity. Thus, there was no use to change the catalyst concentration that have been used until now. Henceforth, the monitoring of the hydrosilylation reaction was carried out with a catalyst concentration of 0.415 mmol·L^{-1}.

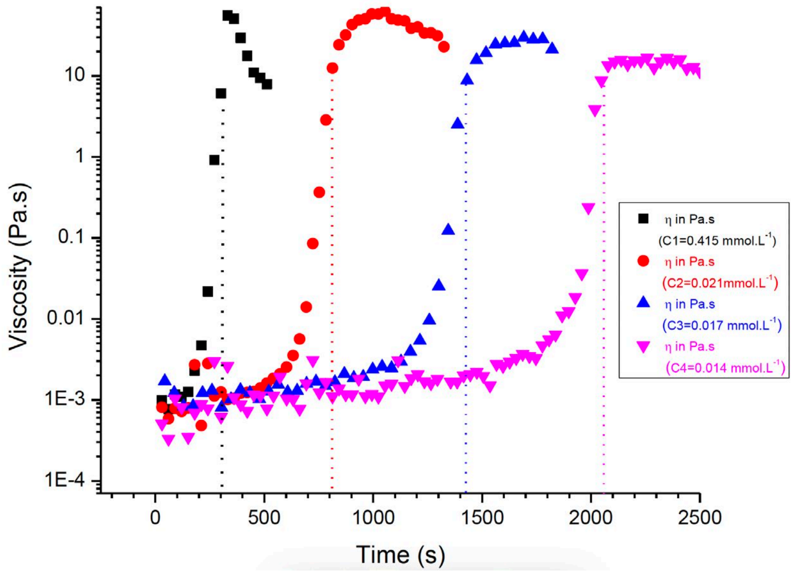

Figure S3. Effect of the concentration of catalyst on the polymerisation reaction. Successive delays of 514 s, 629 s, and 603 s were reported between each catalyst concentration variation, determined for a reached viscosity of roughly 10 Pa s.
